# Point of care echocardiography and lung ultrasound in critically ill patients with COVID-19

**DOI:** 10.1007/s00508-021-01968-y

**Published:** 2021-10-29

**Authors:** Martin Altersberger, Matthias Schneider, Martina Schiller, Christina Binder-Rodriguez, Martin Genger, Mounir Khafaga, Thomas Binder, Helmut Prosch

**Affiliations:** 1 Rehabilitation Center Hochegg for Cardiovascular and Respiratory Diseases, Friedrich Hillegeist Straße 2, 2840 Grimmenstein, Austria; 2 Department of Cardiology, Nephrology and Intensive Care Medicine, State Hospital Steyr, Steyr, Austria; 3 Department of Internal Medicine II, Division of Cardiology, Medical University of Vienna, Vienna, Austria Waehringer Guertel 18–20, 1090; 4 Department of Radiology, State hospital Neunkirchen, Neunkirchen, Austria; 5 Department of Biomedical Imaging and Image-guided Therapy, Medical University of Vienna, Vienna, Austria

**Keywords:** COVID-19, Lung ultrasound, Echocardiography, Point-of-care, POCUS

## Abstract

Hundreds of millions got infected, and millions have died worldwide and still the number of cases is rising.

Chest radiographs and computed tomography (CT) are useful for imaging the lung but their use in infectious diseases is limited due to hygiene and availability.

Lung ultrasound has been shown to be useful in the context of the pandemic, providing clinicians with valuable insights and helping identify complications such as pleural effusion in heart failure or bacterial superinfections. Moreover, lung ultrasound is useful for identifying possible complications of procedures, in particular, pneumothorax.

Associations between coronavirus disease 2019 (COVID-19) and cardiac complications, such as acute myocardial infarction and myocarditis, have been reported. As such, point of care echocardiography as well as a comprehensive approach in later stages of the disease provide important information for optimally diagnosing and treating complications of COVID-19.

In our experience, lung ultrasound in combination with echocardiography, has a great impact on treatment decisions. In the acute state as well as in the follow-up setting after a severe or critical state of COVID-19, ultrasound can be of great impact to monitor the progression and regression of disease.

**Video online:**

The online version of this article contains 4 videos. The article and the videos are available online (10.1007/s00508-021-01968-y). The videos can be found in the article back matter as “Electronic Supplementary Material”.

## Introduction

At the end of December 2019, a novel coronavirus (SARS-CoV-2) began to spread in the city of Wuhan, Hubei Province, China [1, 2]. Now, at the time of writing, the whole world is being held hostage by what has become a global pandemic, and a true burden for healthcare professionals [3]. As of August 2021, almost 205 million cases have been reported worldwide and this number might be underestimated [4–10].

### Clinical presentation and diagnosis of COVID-19

Patients with COVID-19 frequently present with fever and cough but a loss of taste and smell has also been reported [11, 12]. Of COVID-19 positive cases 81% lead to a mild disease, whilst 14% of diagnosed patients become severely ill (requiring hospitalization) and 5% become critically ill [11]. Patients presenting with hypoxia, Glasgow coma scale <15, and a breathing rate >30/min, have a higher mortality rate and should be admitted to hospital [13]. Patients who become critically ill due to acute respiratory failure or acute respiratory distress syndrome (ARDS) need to be treated in an intensive care unit (ICU). Furthermore, bacterial superinfection, cardiac complications, such as arrhythmia or acute myocardial injury (AMI) and iatrogenic complications (pneumothorax), also have to be combatted in an intensive care setting [11, 14–16].

Diagnosing COVID-19 can be challenging. At the beginning of the pandemic, the real-time polymerase chain reaction (RT-PCR) had not been validated. Therefore, false negatives occurred due to inadequate diagnostic timing [17]. Overall, combining molecular diagnostics with thorough clinical examinations and imaging can reduce the risk of false negatives, and allow efficient management in the context of this pandemic [18].

## Role of imaging

Imaging plays a central role for the diagnosis, differential diagnosis and the detection of complications in many diseases. In the context of highly contagious diseases like COVID-19, the potential benefits of imaging have to be balanced against the risk of spreading the infection by exposing personnel during the examination. Consequently, a number of scientific societies have released guidelines on the use of imaging in COVID-19 [19, 20]. Imaging should not be performed in asymptomatic patients or patients with only mild symptoms and should be restricted to patients with moderate to severe symptoms [19]. For the duration of the pandemic and up to the present day, nasopharyngeal RT-PCR testing has been the gold standard for diagnosing COVID-19 infections [10]. The sensitivity of this test is variable, with reported sensitivities ranging from 50% to 98%; however, with optimal patient selection a high sensitivity and specificity can be obtained [18, 21]. Antigen-detecting rapid diagnostic tests (ad-RDT) are easy to use, are less costly than RT-PCR testing and, at 10–30 min, take less time to process but they also have a lower sensitivity. The specificity of ad-RDT tests is reported to be high, at >97%. Ad-RDT testing by trained personnel is recommended as a screening tool to minimize RT-PCR testing, 5–7 days after the onset of symptoms [22].

Point of care ultrasound (POCUS) has been proposed for use as a bed-side imaging modality for outpatient COVID-19 triage [23, 24]. Furthermore, POCUS has been recommended for follow-up care of COVID-19 patients in the ICU, and to identify cardiac pathologies such as myocarditis, acute coronary syndrome (ACS), pulmonary embolism and cardiac tamponade [25–29]. POCUS in the ICU should be performed whenever there is a need to answer a clinical question; for example, when trying to identify the cause for a decline in the respiratory condition of a patient. Overall, the use of POCUS and the advantages thereof need to be a balanced with the workload in the ICU, and the risks of disturbing patients’ positions, especially in hemodynamically unstable patients [30].

Here, we present an ultrasound assessment protocol that can be used on COVID-19 patients who are critically or severely ill. Additionally, it is recommended that this protocol is undertaken at follow-up examinations after COVID-19 infections. Given the growing number of handheld devices and a broad use of POCUS [23], we provide images and ultrasound loops taken with both handheld devices as well as high-end machines.

## Lung ultrasound in COVID-19

### Preset and settings

There are important factors to be considered with respect to the presets and settings of devices used for lung ultrasound (LUS). In order to be able to detect ultrasound artifacts associated with COVID-19 pneumonia, a specific lung preset with a low mechanical index, single-focal point modality and no harmonic imaging or any other cosmetic filters should be chosen [27]. The focal zone should be placed at the pleural line.

### COVID-19 pneumonia

In COVID-19 patients, a linear transducer should be used for scanning the anterior and posterior regions of the chest (at a depth setting of 6–8 cm). Alternatively, a convex transducer can be used, and is particularly useful for scanning obese patients and the lateral or posterior regions of supine patients [23].

**Fig. 1:**
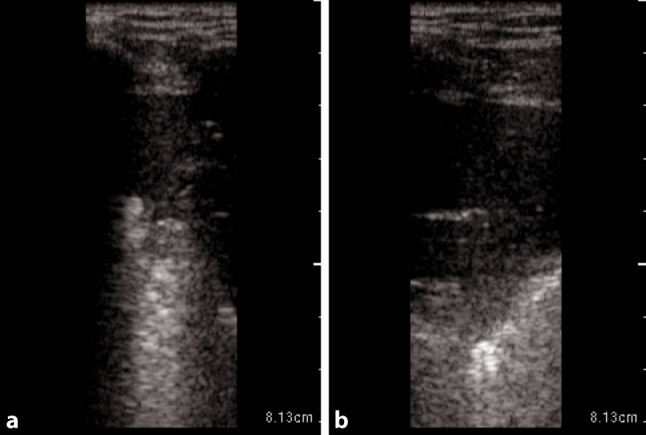
Large anterior consolidation of the lung in POCUS in a longitudinal plane on the right anterior hemithorax in critical COVID-19. **a** and **b** stays the same as the intro provides the information needed in both images

**Fig. 2:**
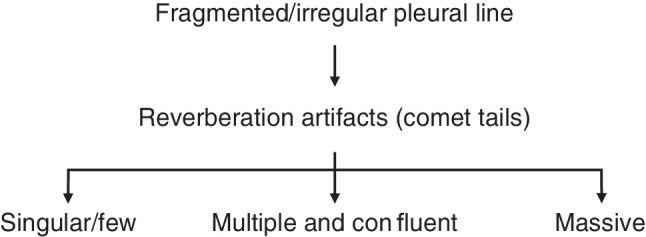
Suggested quantification for reverberation artifacts (comet tails) in COVID-19

**Fig. 3:**
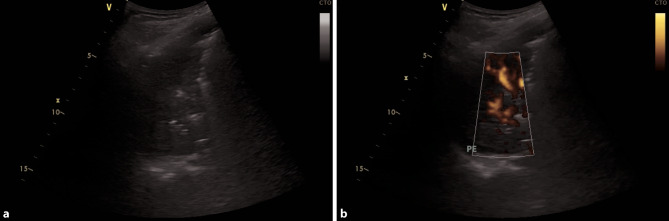
Bacterial superinfection of the left lower lung lobe in COVID-19. **a** Large consolidation with air bronchograms in a bacterial superinfection in COVID-19. **b** Same consolidation with power Doppler to display vascularization, a small pleural effusion is seen (*PE*)

Typical LUS findings in patients with COVID-19 pneumonia are bilateral areas of fragmentation and irregularities of the pleural line, sometimes with massive reverberation artifacts (recently named light beam appearance), small subpleural consolidations, and a reduction or absence of pleural sliding [24]. Reverberation artifacts arising from a fragmented pleural line are indicative of diffuse alveolar damage, diffuse parenchymatous lung diseases, or inflammatory diseases such as COVID-19 [28]. As the disease progresses, consolidations can be seen (Fig. 1). Small consolidations and subpleural consolidations are typical, yet not specific to COVID-19 [26]. As seen on ultrasound images, consolidations can be described as hypoechoic areas with small hyperechoic structures within (bronchograms), in combination with a tissue-like appearance or hepatization of the lungs [31, 32]. If there is a consolidation of an entire lobe, the borders will be well defined, whereas in smaller consolidations, the deeper borders appear irregular, described as the shred sign [33]. As lung ultrasound may help to predict the clinical course and outcomes of patients with COVID-19, and for follow-up purposes, the authors recommend a quantification of reverberation artifacts (Fig. 2), and suspected areas of consolidation (Figs. 1 and 3; Table 1; [24, 34]).

**Table 1:** Suggested measurements—size quantification of consolidation

Consolidation	
Small	0.5 ≤ 2 cm
Moderate	2 ≤ 5 cm
Large	> 5 cm

Dynamic air bronchograms (hyperechoic artifacts moving with the respiratory cycle) are observed within areas of consolidation. Fluid bronchograms (bronchi filled with anechoic fluid within areas of consolidation) present frequently as well. Consolidations can involve entire lobes or whole lungs [24, 35]. Doppler can be used to identify vascularization in consolidations, which is an additional indicator of pneumonia: a tree-like appearance of vessels in color Doppler and a predominately triphasic flow signal in pulsed wave Doppler, can be used to identify pulmonary arteries in consolidations. Monophasic blood flow is found in bronchial arteries. This can be help detect complications such as necrosis, by visualizing hypoechoic areas with no blood flow [36, 37].

**Fig. 4:**
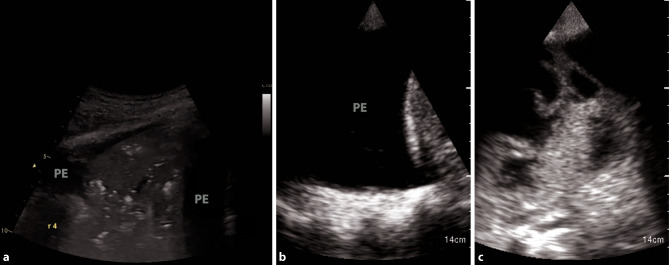
Different types of pleural effusion. **a** Consolidation with tissue-like appearance in pneumonia. *PE* pleural effusion. **b** Large, uncomplicated pleural effusion in heart failure. **c** Complicated pleural effusion, empyema

Overall, pleural effusions in the context of bacterial superinfections tend to be small and localized in patients with COVID-19 [28]. If large pleural effusions are present, other differential diagnoses, such as heart failure (in particular right ventricular failure), kidney failure, and liver cirrhosis, have to be considered (Fig. 4b; [38]).

A pneumonia diagnosis, however, should never be made solely by imaging, but by interpreting the overall clinical status and the history of the patient, as well as laboratory parameters [35].

**Fig. 5:**
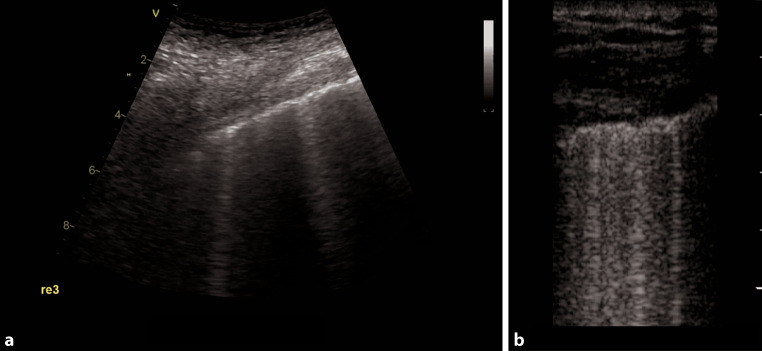
COVID-19 typical artifacts. **a** Fragmented pleural line with comet tail artifacts. **b** Fragmented pleural line with many comet tail artifacts

**Fig. 6:**
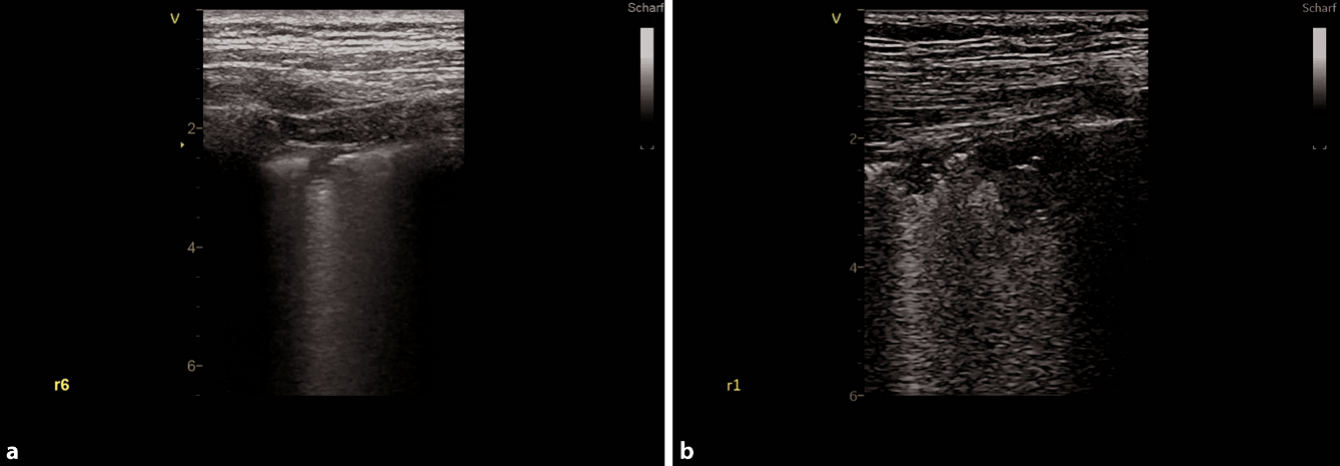
Small consolidations in COVID-19. **a** Small consolidation with comet tail artifact in zone 6, right hemithorax. **b** Two small consolidations in zone 1, right hemithorax

**Fig. 7:**
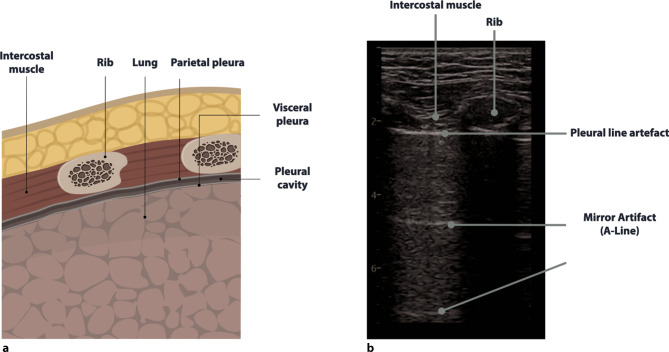
Anatomy and sonoanatomy of the lung. **a** Anatomy of the lung in a longitudinal view. **b** Normal sonographic anatomy in a patient after a severe COVID-19 infection

**Fig. 8:**
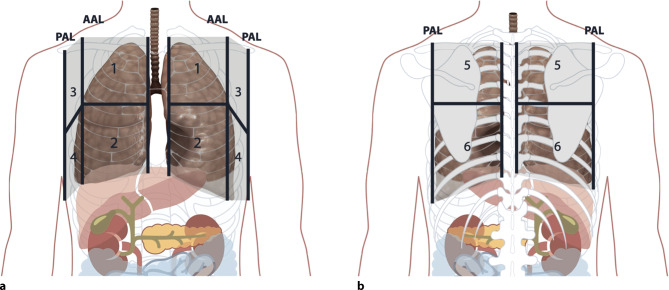
Anatomical landmarks and scanning zones of lung ultrasound. **a** Anterior scanning zones of the chest. *AAL* anterior axillary line, *PAL* posterior axially line. **b** Posterior scanning zones of the chest

As COVID-19 pneumonia is characterized by a patchy distribution of affected lung parenchyma, areas with a normal appearance in LUS are seen next to pathological areas (Figs. 5a, 6 and 7; [24]). Due to the patchy distribution, a thorough examination is recommended, yet there is no validated scanning scheme [23]. We recommend a 12-zone scanning protocol, as it is, in our experience, simple to apply, reproducible, and easy to learn (Fig. 8). Beginning with zone one with a longitudinal view, followed by a transverse view, a scan of all intercostal spaces should be performed. Whenever possible, scanning of the posterior regions should be included for patients in a supine position, although this may not always be feasible. In this case, it is recommended to consider scanning the posterior regions during prone positioning [23].

### Diagnosis of pulmonary complications

POCUS has been demonstrated to be a valuable tool to diagnose complications of COVID-19, such as pneumothorax or pleural effusions, or to confirm the suspected complications such as bacterial superinfections (Fig. 4a, c).

**Fig. 9:**
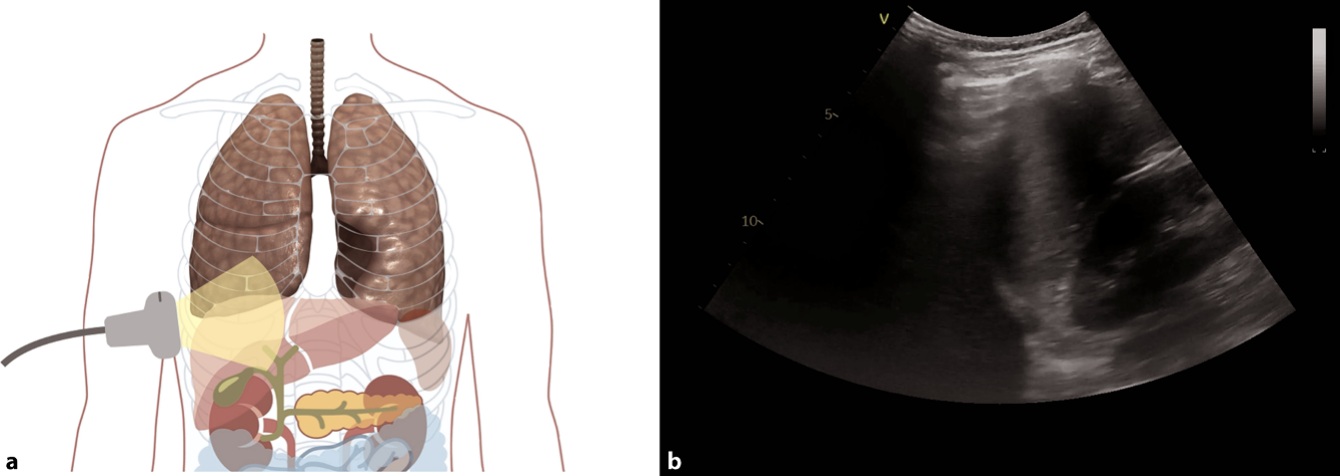
Evaluation for pleural effusion—right hemithorax. **a** Schematic of the right sided approach. **b** Normal sonographic anatomy without pleural effusion

**Fig. 10:**
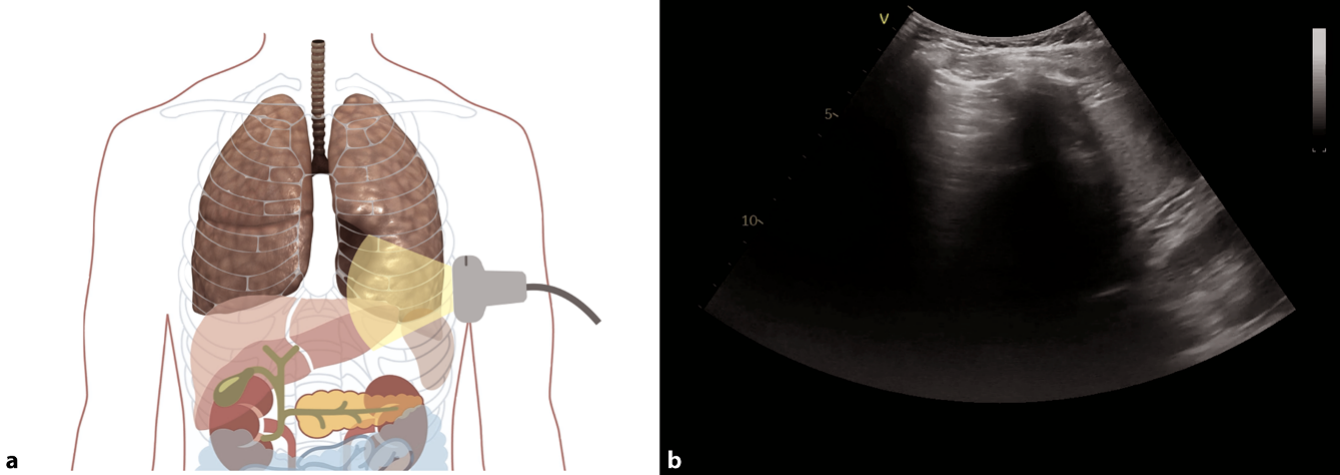
Evaluation for pleural effusion—left hemithorax. **a** Schematic of the left-sided approach. **b** Normal sonographic anatomy without pleural effusion

Pleural effusions are reportedly rare in COVID-19 pneumonia, and thus indicate a bacterial superinfection or heart failure [26]. To detect pleural effusions in supine patients, e.g. in an ICU, we recommend scanning the posterior regions with a convex or cardiac transducer as posterior as possible (Figs. 9 and 10). In an upright patient, the posterior regions—zone six (Fig. 8)—can be visualized with a convex or cardiac transducer to identify pleural effusions. In large pleural effusions, compression atelectasis can be visualized. Likewise, as previously mentioned, differential diagnoses of pleural effusions have to be considered [39]. The suspicion of heart failure is further supported by the presence of reverberation artifacts, which arise from a non-fragmented pleural line (B-lines). These B‑lines arise from the smooth pleural line in a bilateral homogenous way, not fading until the end of the screen, moving with pleural sliding and erasing other artifacts (A-lines). The presence and quantity of B‑lines correlate with the severity of clinical presentation of patients [24, 35, 40].

Patients who are critically ill often need to undergo procedures, for many of which a pneumothorax is a potential complication [15, 16]. In COVID-19, there are also cases with spontaneous pneumothorax described, making this entity an important differential diagnosis of acute respiratory distress [41]. In ultrasound, an easy protocol for detecting a pneumothorax can be applied [40]. Scanning should start at the highest point of the thorax, identifying whether lung sliding is present. Lung sliding corresponds to the motion of the parietal and visceral pleura. In a pneumothorax, there is gas between the parietal and visceral pleura, and pleural sliding is lost. The presence of pleural sliding rules out a pneumothorax [32]. In cases where there is an absence of lung sliding but a presence of vertical reverberation artifacts (B-lines, comet tails), a pneumothorax can be ruled out as well, as there has to be a connection between the parietal and visceral pleura to create such artifacts [32]. The presence of a lung pulse (a movement of the pleural line which is synchronous with the cardiac rhythm) rules out pneumothorax as well [42]. The most reliable rule in diagnosing a pneumothorax is the so-called lung point sign—the transition zone between the pneumothorax and the aerated lung (sliding on one side of the screen lung can be identified, whilst on the other, no lung sliding is present, video 2—lung point sign) [40]. It is recommended to use a point of care approach, performing a scan of the most anterior region (mostly in between zones 1 and 2) in a supine positioned patient [40].

## Indications—cardiac ultrasound

In cardiac ultrasound, standard protocols exist, which should be followed in a comprehensive examination. In a POCUS setting, the aim of the examination is to visualize the heart and to detect specific pathologies such as a pericardial effusion, tamponade, severely reduced left and right ventricular functions, or hypovolemia. Decisions may need to be made as to whether a referral to a comprehensive cardiac ultrasound examination or for other tests is indicated [29, 43].

In COVID-19, echocardiography is useful to evaluate the heart for myocarditis, myocardial injury, acute coronary syndromes and pulmonary embolism [44, 45].

**Fig. 11:**
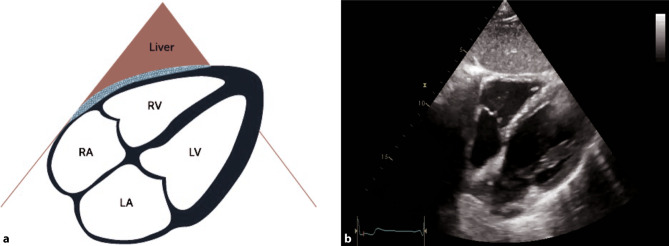
Subcostal 4‑chamber view (*4‑ChV*). **a** Schematic of a subcostal 4‑ChV. *RV* right ventricle, *RA* right atrium, *LV* left ventricle, *LA* left atrium. **b** 2D image of a subcostal 4‑ChV

**Fig. 12:**
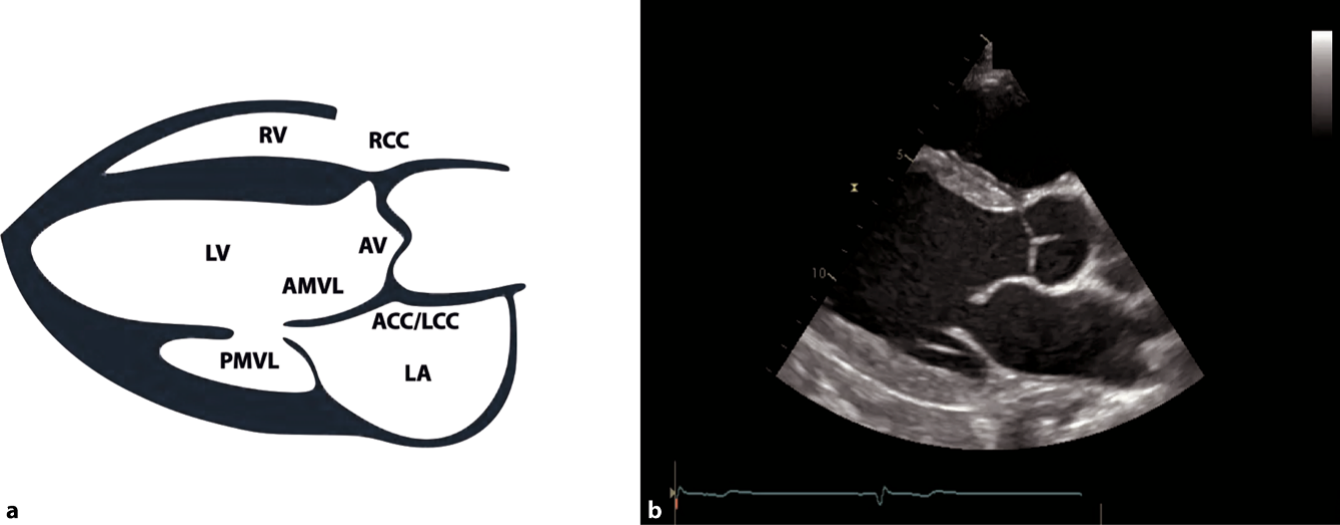
Parasternal long axis view (*PLAX*). **a** PLAX-left ventricle = *LV*. *AMVL* anterior mitral valve leaflet, *PMVL* posterior MVL, *AV* aortic valve, *RCC* right coronary cusp, *ACC/LCC* non-coronary/left coronary cusp, *RV* right ventricle, *LA* left atrium. **b** 2D image of a PLAX view

**Fig. 13:**
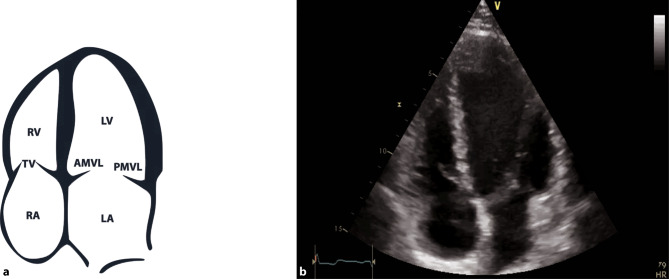
Apical 4‑chamber view (*4‑ChV*). **a** Schematic of a 4-ChV. *RV* right ventricle, *TV* tricuspid valve, *RA* right atrium, *LV* left ventricle, *LA* left atrium, *AMVL* anterior mitral valve leaflet, *PMVL* posterior mitral valve leaflet. **b** 2D image of an apical 4‑ChV

A comprehensive examination can be achieved with various views; however, in acute and intensive care settings, a subcostal 4‑chamber view (subcostal 4‑ChV) should be utilized as it provides a lot of information and is easiest to achieve in a supine patient as well. If imaging from a subcostal approach is not possible, alternative views can be chosen, such as a parasternal long axis view (PLAX) or an apical 4‑chamber view (4-ChV) (Figs. 11, 12 and 13).

### Myocardial injury and perimyocarditis/myopericarditis

Patients with COVID-19 pneumonia can develop myocardial injury or even fulminant myocarditis [44, 45]. Echo findings in myocarditis include chamber size alterations, increasing wall thickness, global or regional ventricular dysfunction, and diastolic dysfunction [46]. In pericarditis, a pericardial effusion can be detected [47]. It can mimic ischemic cardiomyopathy [48]. In fulminant myocarditis, a nondilated, thickened and hypocontractile left ventricle is seen, as the inflammatory response results in interstitial edema and loss of ventricular contractility (video 4—myocardial edema of the left ventricle (LV) in myocarditis) [49].

**Fig. 14:**
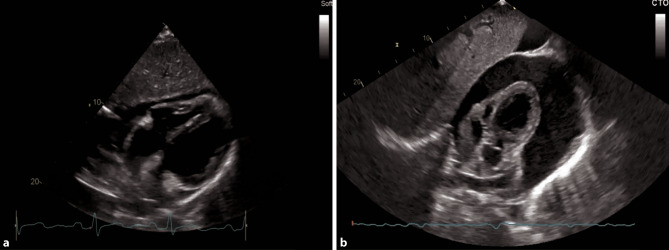
Pericardial effusion in a subcostal 4‑ChV. **a** Small-medium pericardial effusion. **b** Large pericardial effusion

For detecting myocarditis in an intensive care setting, a subcostal 4‑ChV should initially be chosen (Fig. 11). In a subcostal approach, pericardial effusion can also be visualized (Fig. 14). An assessment of the global, left and right ventricular function is also possible. An alternative view to detect pericardial effusion and reduced global ventricular function is the 4‑ChV. Regional wall motion abnormalities (WMA) should be assessed in a comprehensive examination [43].

In our experience, hyperdynamic right ventricular function (RVF) and left ventricular function (LVF) are frequently seen in combination with mildly elevated systolic pulmonary artery pressures (sPAP), remaining after intensive care treatment in patients, with or without prior cardiovascular disease such as hypertension; however, a normalization can be seen after a few months in a majority of patients.

In strain imaging, there can be regional and global changes, which persist over the course of several months. The implications for treatment over a longer period and the clinical relevance in the follow-up have not yet been established [50].

### Pulmonary embolism

Hypercoagulopathy is described in COVID-19 patients, and pulmonary embolism is a life-threatening complication thereof. Therefore, it is crucial to recognize the echocardiographic features of a hemodynamically relevant pulmonary embolism, to be able to initiate treatment [28, 38, 51].

**Fig. 15:**
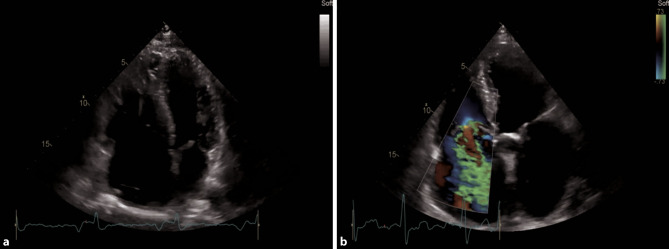
Pathologies of the right heart in an apical 4‑ChV. **a** RV dilatation due to myocarditis. **b** Severe tricuspid regurgitation

In an acute, hemodynamically relevant pulmonary embolism (PE), the main features in echocardiography are a dilated right ventricle (RV), with the basal diameter >41 mm (as seen in Fig. 15a), reduced RVF, especially at the basal parts, S’ <9.5 cm/s, tricuspid annular plane systolic excursion (TAPSE) <17 mm, sometimes with a hyperdynamic apical region (McConnell sign). The sPAP is normal, low or mildly elevated [52]. A 60/60 sign which describes an sPAP of more than 30 mm Hg but less than 60 mm Hg, and a right ventricular outflow tract acceleration time of less than 60 ms, can be present [53]. The inferior vena cava (IVC) can be dilated with reduced, or even without, respiratory collapsibility. In a parasternal short axis view, a D-shape of the left ventricle can be visualized, and severe tricuspid regurgitation (TR) can be present (Fig. 15b; [54, 55]). None of these echocardiographic findings are specific for acute PE, but in the setting of COVID-19, in particular in an intensive care setting, it is reported that up to one third of patients do develop PE [56].

On the ICU, the optimal view to visualize the right heart is a subcostal 4‑ChV, permitting visual assessment of RVF and size. Color Doppler of the region of the tricuspid valve can provide information as to whether severe TR is present (Fig. 15b). Additional views, especially in follow up examinations, include apical and parasternal views, as seen in Figs. 12 and 13. SPAP should be measured with continuous wave Doppler across the tricuspid valve, according to current guidelines [57].

### Acute coronary syndromes and heart failure

Acute heart failure can be the primary symptom of COVID-19 infection with 50% of COVID-19 patients having no prior cardiovascular diseases [44]. Severe inflammation increases the risk of acute myocardial infarction [58].

Detecting regional WMA in ICU patients can be challenging. Several views should be obtained. In an apical 4‑ChV, the apical regions can be visualized, and in the PLAX, the posterolateral and the anteroseptal wall of the LV can be seen. For evaluation of RV dysfunction and larger LV infarcts, a subcostal 4‑ChV is optimal [43, 57]. For an evaluation of regional WMA and RVF, a comprehensive transthoracic examination is required [43].

## Conclusion

In COVID-19, ultrasound plays an important role in diagnosing pneumonia, corresponding complications, including ACS and bacterial superinfections, and cardiac conditions [23, 44, 45].

Lung ultrasound has the potential to be useful in diagnosing COVID-19 pneumonia and complications such as bacterial superinfection or pneumothorax [24]. The need for other imaging modalities can be reduced, and radiation can be avoided [59]. Lung ultrasound can aid treatment of patients in a critical condition and in time help identify complete regression of the disease [60, 61].

We recommend following a standard 12-zone protocol for LUS in a COVID-19 setting, whenever possible, with the aim of identifying changes over time, using the provided measurements and tools for quantification. In detecting a pneumothorax, a POCUS approach should be chosen [23, 40]. Automated measurements for reverberation artifacts can be used and might detect subvisible biomarkers helping to differentiate the cause of reverberation artifacts and might be useful in the future [62, 63].

Echocardiography in a point of care approach is an easy and accessible tool to optimally identify cardiac complications in COVID-19, such as tamponade, pericardial effusion and severely reduced LV and RV function [44, 45]. In a comprehensive approach, we recommend following the internationally established protocols and include strain analysis to monitor patients after COVID-19 to identify persistent changes in echocardiography such as elevation of sPAP, regional WMA, reduction in global longitudinal strain (GLS) of the LV, RV dilatation and diastolic dysfunction [50, 57, 64].

Overall, echocardiography and lung ultrasound combined can be of tremendous help in identifying life-threatening conditions and can be used in the management of patients in intensive care units. It is also a valuable tool for in follow-up care in the context of the pandemic [50, 65]. Especially the topic of strain imaging with the first evaluation on the ICU of left and right ventricular strain as well as in the follow-up will be relevant in the future as a reduced GLS and reduced RV strain seem to reflect a marker of poor prognosis [66, 67].

## Supplementary Information

Video 1: Lung ultrasound and CT imaging. Left: baseline with LUS and CT imaging after intensive care treatment—red circle indicating subpleural consolidation. Right: follow-up LUS and CT imaging after 4 months, showing a reduction in the area of consolidation. In CT-imaging, almost no residual findings of COVID-19 are present

Video 2: Lung point sign in pneumothorax

Video 3: Left ventricular strain imaging in COVID-19. Left: reduction in strain at the basal parts with normal GLPSS. Right: hyperdynamic GLPSS

Video 4: Myocardial edema in myocarditis

## Abbreviations

4‑ChV: Apical 4‑chamber-view
ACS: Acute coronary syndrome
ad-RDT: Antigen-detecting rapid diagnostic tests
AMI: Acute myocardial injury
ARDS: Acute respiratory distress syndrome
GLS: Global longitudinal strain
ICU: Intensive care unit
IVC: Inferior vena cava
LUS: Lung ultrasound
LV: Left ventricle
LVF: Left ventricular function
PE: Pulmonary embolism
PLAX: Parasternal long axis view
POCUS: Point of care ultrasound
RT-PCR: Real-time polymerase chain reaction
RV: Right ventricle
RVF: Right ventricular function
sPAP: Systolic pulmonary artery pressure
Subcostal 4‑ChV: Subcostal 4‑chamber view
TAPSE: Tricuspid annular plane systolic excursion
TR: Tricuspid regurgitation
WMA: Wall motion abnormalities
